# Ecological and Genetic Differences between *Cacopsylla melanoneura* (Hemiptera, Psyllidae) Populations Reveal Species Host Plant Preference

**DOI:** 10.1371/journal.pone.0069663

**Published:** 2013-07-16

**Authors:** Valeria Malagnini, Federico Pedrazzoli, Chiara Papetti, Christian Cainelli, Rosaly Zasso, Valeria Gualandri, Alberto Pozzebon, Claudio Ioriatti

**Affiliations:** 1 Centre for Technology Transfer, FEM-IASMA, San Michele all’Adige (TN), Italy; 2 Department of Biology, University of Padua, Padova, Italy; 3 Department of Agronomy, Food, Natural Resources, Animals and Environment, University of Padua, AGRIPOLIS, Legnaro (PD), Italy; Natural Resources Canada, Canada

## Abstract

The psyllid *Cacopsylla melanoneura* is considered one of the vectors of ‘*Candidatus* Phytoplasma mali’, the causal agent of apple proliferation disease. In Northern Italy, overwintered *C. melanoneura* adults reach apple and hawthorn around the end of January. Nymph development takes place between March and the end of April. The new generation adults migrate onto conifers around mid-June and come back to the host plant species after overwintering. In this study we investigated behavioural differences, genetic differentiation and gene flow between samples of *C. melanoneura* collected from the two different host plants. Further analyses were performed on some samples collected from conifers. To assess the ecological differences, host-switching experiments were conducted on *C. melanoneura* samples collected from apple and hawthorn. Furthermore, the genetic structure of the samples was studied by genotyping microsatellite markers. The examined *C. melanoneura* samples performed better on their native host plant species. This was verified in terms of oviposition and development of the offspring. Data resulting from microsatellite analysis indicated a low, but statistically significant difference between collected-from-apple and hawthorn samples. In conclusion, both ecological and genetic results indicate a differentiation between *C. melanoneura* samples associated with the two host plants.

## Introduction

The agronomic importance of the Hemiptera genus *Cacopsylla* is due to the role that several of its species play in the transmission of phytoplasma diseases belonging to the apple proliferation cluster, including ‘*Candidatus* Phytoplasma mali’, ‘*Ca*. P. pyri’ and ‘*Ca*. P. prunorum’ [1]. ‘*Ca.* P. mali’ is the etiological agent of apple proliferation (AP) disease, which is a severe problem in Italian apple (*Malus domestica* Borkh.) orchards. The economic impact of the disease is quite high: besides symptoms on shoots and leaves, such as witches’ brooms, enlarged stipules and early leaf reddening, the disease causes a reduction in size (up to 50%), weight (by 63–74%) and, therefore, quality of fruits [2]. In the last ten years, due to the epidemic spread of the AP disease, 6,000 ha of apple orchards were uprooted and replanted in Trentino region.

*Cacopsylla melanoneura* (Förster), one of the most common psyllids in Italian apple orchards, is known as a vector of AP in Northwestern Italy [3], while it was shown not to transmit this disease in Germany and neighbouring countries [4]. This univoltine species is linked to some *Rosaceae Maloideae*, such as *Crataegus*, *Malus* and *Pyrus* spp. In Italy, the biological cycle of *C. melanoneura* on apple was studied and described by Tedeschi et al. [3] and Mattedi et al. [5]. In Trentino, overwintered adults reach the orchards when the average of the maximum temperatures of 7 days is above 9.5°C [6], which usually corresponds to the end of January. After mating, *C. melanoneura* females oviposit between the beginning of March and the beginning of April. Neanids hatch in the middle of March and complete their development by the end of April. The adults of the next generation (emigrants) leave the orchard around mid-June and move to the overwintering host plants. Conifers have been reported to be shelter plants for the aestivation and overwintering of the new generation [7]–[9].

Besides apple, the AP agent can also infect other plants, such as other rosaceous fruit trees and woody plants, including hawthorn (*Crataegus monogyna* Jacq.), on which it causes yellowing and/or decline symptoms [10]. For this reason, hawthorn may represent an alternative phytoplasma reservoir for the psyllids, if the insects are able to move from these plants to apple trees. Recently, individuals of *C. melanoneura* collected in nortwestern Italy from hawthorn were found to carry AP-group phytoplasmas, such as ‘*Ca.* P. mali’ and ‘*Ca*. P. pyri’ [11], [12].

Despite the economic importance of this species, little is known about its behavioural aspects, genetic structure, patterns of dispersal at the local and regional scale, and in relation to the host plants. Intrinsic insect characteristics (such as the adult flight capacity), as well as ecological factors related to habitat (i.e. host plant and geographical isolation), may shape the genetic architecture of traits in insect populations [4]. In addition, the existence of host races can also affect gene flow and genetic differentiation in insects [13]. Moreover, especially in agroecosystems, also anthropogenic factors, such as pest management [14], can further contribute to insect population genetic differentiation.

This study was aimed at verifying the hypothesis that the different host plant choice could affect the survival and reproductive performance and shape the genetic structure of *C. melanoneura* samples collected from apple trees and hawthorn bushes, respectively. The ecological effect of the different plant hosting was assessed by a host-switching experiment, while genetic differences between these samples were investigated by genotyping 7 microsatellite markers specifically developed for *C. melanoneura* [15].

## Materials and Methods

### Ethic Statement

All the insects used in the experiments were collected and treated ethically. The individuals used for the analyses were frozen at −80°C to minimize suffering. This study did not involve endangered or protected species and therefore no specific permissions were required for collecting *C. melanoneura* individuals. The collection of insect specimens in private orchards was carried out after obtaining the permission of the owners.

### Sampling

In this study “samples” are defined as groups of individuals of *C. melanoneura* collected from the same plants in a specific locality.

The samples analyzed were collected in apple orchards or hawthorn (*C. monogyna* Jacq.) hedgerows in Italy (Trentino-Alto Adige and Aosta Valley), Southern Germany and France (only from apple plants). Some other psyllids were collected from their shelter plants [conifers such as *Picea abies* (L.) H. Karst. and *Pinus mugo* Turra] in Northeastern Italy and France. Sampling details are reported in Table 1. Samples were collected by sweep-netting between December (from conifers) and the end of March (from apple and hawthorn).

**Table 1:** *Cacopsylla melanoneura* sampling.

Host plant	Locality	Acronym	Coordinates (lat. and long.)	Altitude (m)	Sample size (N)
Hawthorn	Cles (TN-Italy)	HaCL	N 46°21′E 11°02′	674	22
	Maso Parti (TN-Italy)	HaMP	N 46°11′E 11°06′	204	30
	Rumo (TN-Italy)	HaRU	N 46°26′E 11°01′	953	41
	Chambave (AO-Italy)	HaCH	N 45°44′E 07°33′	723	42
	Neustadt (Germany)	HaNE	N 49°21′E 08°08′	150	48
Apple	Borgo Valsugana (TN-Itay)	ApBO	N 46°02′E 11°28′	481	48
	Oltrecastello (TN-Italy)	ApOL	N 46°04′E 11°09′	377	26
	San Michele (TN-Italy)	ApSM	N 46°11′E 11°08′	291	41
	Vervò (TN-Italy)	ApVE	N 46°18′E 11°07′	766	28
	Vigalzano (TN-Italy)	ApVI	N 46°04′E 11°13′	512	40
	Aosta (AO-Italy)	ApAO	N 45°44′E 07°18′	577	36
	Meckenheim (Germany)	ApME	N 49°24′E 08°14′	116	49
	Stotzheim (France)	ApST	N 48°38′E 07°49′	138	44
Conifers	Sopramonte (TN-Italy)	CoSO	N 46°04′E 11°03′	613	40
	Vason (TN-Italy)	CoVA	N 46°02′E 11°03′	1643	23
	La Grave (Hesperault-France)	CoES	N 43°58′E 03°22′	730	19

Host plant, geographical collection sites, acronyms, coordinates and sample sizes of *Cacopsylla melanoneura* samples are reported. Localities are shown in Figure 4.

### Species Determination

*C. melanoneura* is often mistaken on hawthorn and conifers for another species, *C. affinis* (Löw), which is morphologically very similar [12]. Only males of the two species can be distinguished by examining terminalia, following Ossiannilsson keys [8], while females are identical. For this reason, at the end of behavioural experiments and before genetic analyses species identifications were verified by specific amplifications with the primers MEL_fw/MEL_rev, which amplify only *C. melanoneura* individuals, and AFF_fw/AFF_rev, specific for *C. affinis*, as described in [16]. These primer pairs amplify a species-specific segment of the control region of the mitochondrial genome;.the PCR products were visualized in an agorose gel (1%), stained with SYBR®Safe (Life Technologies, Grand Island, NY, USA). The total DNA was extracted following Doyle and Doyle method [17], the sequences of the primers and the annealing temperatures are reported in Table 2.

**Table 2:** Summary data for the microsatellites developed from *Cacopsylla melanoneura.*

Name	Primer sequences (5′-3′)	*T*a (°C)	GenBank accession no.	Reference
AFF_fw	TTTAACCACCTCAAACTCAA	55		[16]
AFF_rev	CGTAAAATTCTTGGCGA			
MEL_fw	TTTTATCCACTCTTAAAGCTTG	55		[16]
MEL_rev	TGATAGAGCTTTTTGAATTCTC			
Co03	F: TCTGCACGCAATACCAGAAC	60	DQ414790	[15]
	R: CGCTACATGACGTGTTGTCC			
Co04	F: GGATAGCATCCACATTCCAC	60	DQ414791	[15]
	R: CCTCTTTAGGACACGGACTTG			
Co11	F: TTGAATTCTTGAACCTCTGACC	56	DQ631795	[15]
	R: TCACAAATGGAGCTTACAGGTG			
Co12	F: GCTCTTTCTCAATCCGTCCTG	60	DQ414793	[15]
	R: GAGGTGAGAGGGCGGAATAC			
Co13	F: TAAGAAGTTAGAAAGGGAGGGT	56	DQ631796	[15]
	R: GGGTCGGATTTTGGAAACAG			
Co14	F: ACAACACATGGCCCATATTTAC	56	DQ631797	[15]
	R: CTCAGTGGTGTGAATCTGACG			
Co18	F: TTTTGTTTGTTTTAGTGTTCATCCTC	53	DQ414794	[15]
	R: ACTAGGTCGGGGGTGATGTC			

Locus name, primer sequences, annealing temperature (*T*_a_), the GenBank accession number and the reference.

### Host-switching Experiments

The effect of different host plants choice on two populations of *C. melanoneura* collected from different host plants was evaluated in terms of survival and reproductive performance. One population of *C. melanoneura* (ApOL) was collected from apple trees in Oltrecastello (Trento - TN) and one from hawthorn bushes (HaCL) in Cles (TN). The distance between the two localities is about 40 km. In two bi-factorial laboratory experiments, the native host plants (apple and hawthorn) and potential host plants (hawthorn and apple, respectively) were considered as experimental factors. The initial experiment involved 80 overwintered adult pairs, 40 collected from apple trees and 40 from hawthorn bushes in Trentino at the end of March 2007. The experimental design consisted of four treatments of 20 replicates. Each treatment was constituted by one population×host plant combination (ApOL×Hawthorn, ApOL×Apple, HaCL×Apple, HaCL×Hawthorn), each replicate corresponding to a shoot. Survival and oviposition of females on different host plants were compared by isolating one overwintering female and one male on the shoot. Each shoot was placed in a glass tube (diameter 3 cm, height 16 cm), inserted into a green sponge soaked with Murashige-Skoog (MS) nutritive solution [18], and kept in a growth chamber under controlled conditions (20°C with a 16∶8 h photoperiod). The shoots were replaced every two days and *C. melanoneura* couples were gently transferred to the new shoots with a thin paintbrush. The survival time of adult females, number of eggs laid and hatching rate were recorded every two to three days. Male survival was not taken into consideration in the analysis because they were let onto the shoot for mating and then removed.

Survival to adulthood was evaluated by transferring with a fine paintbrush six newly emerged nymphs from the shoots used in host-switching experiments to small apple and hawthorn plants. Six replicates for each treatment were considered. Shoots were planted in plastic pots (10 cm diameter) and kept in plexiglas cylindrical vessels (diameter 10.5 cm, height 27 cm) under controlled conditions (20°C with a 16∶8 h photoperiod).

### Host-switching Data Analysis

The survival rates of the two female populations observed on the different host plants were analyzed applying the LIFEREG procedure of SAS [19] and fitting a Weibull model to survival time. The median insect life spans for the different “Sample×Host plant” combinations (ApOL×Apple, ApOL×Hawthorn, HaCL×Apple, HaCL×Hawthorn) were also estimated. The differences related to population, host plant and their interactions were compared using a Wald chi-square test (α = 0.05) [20]. Oviposition was analyzed by fitting the cumulative number of eggs laid during the experiments to a generalized linear Poisson model with a log-link function, using the GENMOD procedure of SAS Institute [19] and estimating the least-squares means. The Likelihood ratio chi-square test *G^2^* (α = 0.05) was applied to compare the differences related to population, potential host plant and their interactions, while the differences among the least-squares means were evaluated using a Wald chi-square test (α = 0.05). Data on egg-hatching (number of immature insects/number of eggs) and survival to adulthood (number of adults/number of newly emerged nymphs) were analyzed by applying a binomial model with a logit-link function, using the GENMOD procedure of SAS Institute [19]. The Likelihood ratio chi-square test *G^2^* (α = 0.05) was performed to compare the differences related to population, potential host plant and their interactions, while the differences among the least-square means were evaluated with a Wald chi-square test (α = 0.05).

### Population Genetics Experiments and Statistical Analyses

#### DNA extraction

Individuals sampled for genetic analyses were immediately frozen at −80°C after collection, lyophilized and homogenized. Samples were then stored at −80°C until the genomic DNA extraction. Total genomic DNA was extracted from single adult specimens following Doyle and Doyle method [17].

#### Microsatellite genotyping

577 individuals were genotyped for seven microsatellite loci (shown in Table 2) with the procedure described in Malagnini et al. [15]. Genotypes were obtained using an ABI 3100 sequencer (GeneScan-500 ROX as internal standard; Applied Biosystems, Foster City, CA, USA). Allele sizing was performed using GENESCAN ver. 3.1.2 and GENEMAPPER ver. 4.0 (both programs from Applied Biosystems, Foster City, CA, USA). Automated binning was performed using FLEXIBIN ver. 2.0 [21], in order to reduce human-related scoring errors [21]. Moreover, the genotype calling was carried out by two independent people. The presence of genotyping artefacts was checked by (i) re-amplifying and scoring a random sub-sample of individuals, (ii) testing for null alleles, stuttering and large allele drop-out using MICROCHECKER ver. 2.2.3 [22] and (iii) subsequently correcting results for loci with null alleles using FREENA [23]. All the analyses described below were performed with both datasets (the original one and the FREENA-corrected one) and the results obtained were compared to assess whether there were significant differences.

#### Genetic diversity, Hardy-Weinberg equilibrium and linkage disequilibrium

Descriptive statistics such as range of allele sizes (*S*_R_) in a base pair (bp), number of alleles (*N*_a_) and allelic richness (*A*_R_) were calculated using FSTAT ver. 2.9.3.2 [24]. Observed (*H*_O_) and expected (*H*_E_) heterozygosity were calculated using GENETIX ver. 4.05.2 [25]. Tests for conformity with Hardy-Weinberg equilibrium (HWE) and linkage disequilibrium between pairs of loci in each population were performed using the online version of GENEPOP [26].

Significance levels for multiple comparisons were adjusted the standard Bonferroni technique [27], [28].

#### Population structure

In a first evaluation only samples collected from apple and hawthorn of Trentino region were considered; in a second evaluation all samples were included in the analysis. FSTAT ver. 2.9.3.2 [24] was used to compute the overall and population pair-wise *F*_ST_ values. The 95% confidence intervals were estimated using 1,000 bootstrap replicates over the loci and probability values were determined using 1,000 permutations. The nominal significance level was set at α = 0.05. Statistical significance level for multiple comparisons was adjusted using a standard Bonferroni as described above. To provide a better visualization of population samples relationship, a matrix of Slatkin’s linearized *F*_ST_ values [29] for all population sample pairs was obtained with ARLEQUIN ver. 3.5 software [30] and used to produce a Neighbour-Joining [31] unrooted population tree with MEGA ver. 5.0. [32]. The molecular variance analysis (AMOVA) was performed using the ARLEQUIN ver. 3.5 software [30] to test genetic differentiation among and within groups. In a first evaluation, the analysis was carried out according to a model structure in which the eight samples of Trentino region were divided into two groups: one collected from apple (ApBO, ApOL, ApSM, ApVE and ApVI) and the other collected from hawthorn (HaCL, HaMP, HaRU). In a second analysis, we included also samples from Aosta Valley (ApAO and HaCH), Germany (ApME and HaNE) and France (ApST).

Analysis of the population structure was performed with STRUCTURE ver. 2.3.3 software [30] to infer the most likely number of populations (*K*), representative of the whole data set, without the use of any *a priori* information. Ten independent runs of structure were performed for each *K* value from 1 to 9. Each run consisted of a burn-in period of 100,000 steps, followed by 1,000,000 Monte Carlo Markov Chain replicates, assuming an admixture model and correlated allele frequencies. The most likely *K* was chosen comparing the average estimates of the likelihood of the data, ln[Pr(X/K)], for each value of *K* [33], as well as calculating *ad hoc* statistics Δ*K* values (Evanno’s method) [34]. The value of ln[Pr(X/K)] and Δ*K* were obtained by Structure Harvester ver. 0.6.1 [35]. The proportions of membership of each individual in each cluster were also calculated. Also in this case, a first analysis was performed using the Trentino data set including only collected-from-apple and hawthorn samples. Ten independent runs of structure were performed for each *K* value from 1 to 9. A second analysis was carried out using the whole data set. In this case, 10 independent runs were performed for each *K* value from 1 to 17.

## Results

### Species Determination

The correct species assessment was confirmed for all male analysed in this study by both morphological and molecular (PCR) analyses. Genetic analyses carried out on females highlighted that only few individuals belonged to the species *C. affinis* and were present within some collected-from-hawthorn samples. These individuals were excluded from the dataset (data not shown).

### Ecological Experiments and Data Analysis

**Figure 1:**
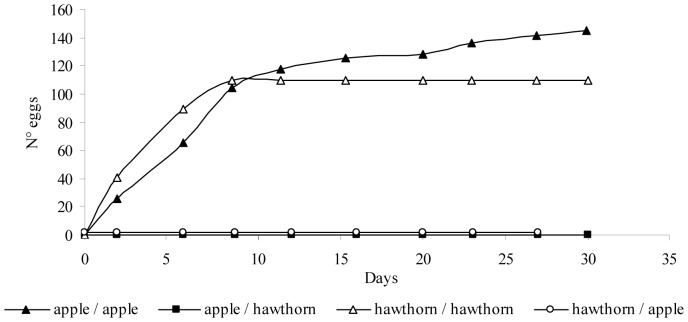
Oviposition of *Cacopsylla melanoneura*. Mean cumulative numbers (untransformed data) of eggs laid by the two samples of *Cacopsylla melanoneura* on the two host plants.

**Figure 2:**
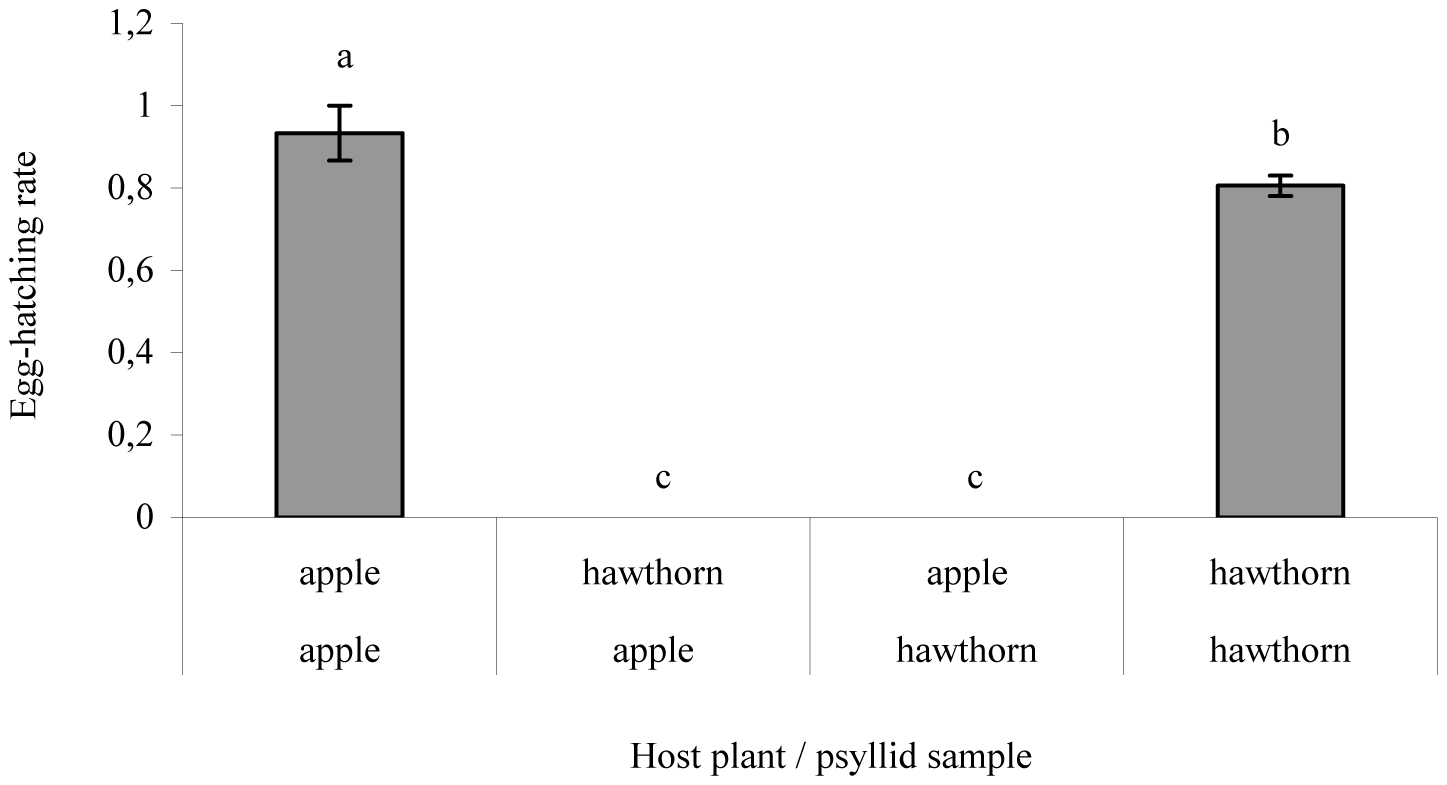
Egg hatching of *Cacopsylla melanoneura*. Egg-hatching rates of the two samples of *Cacopsylla melanoneura* on the different host plants. Different letters indicate significant differences according to the Wald chi-square test (α = 0.05).

Survival analysis detected significant differences between the treatments ApOL×Hawthorn and HaCL×Apple (Wald χ^2^ = 6.34; df = 1; *P* = 0.012), but not between the two treatments ApOL×Apple and HaCL×Hawthorn (Wald χ^2^ = 0.05; df = 1; *P* = 0.828). The analysis found a significant “Sample×Host plant” treatment (Wald χ^2^ = 5.30; df = 1; *P* = 0.021). The survival rate of the collected-from-apple sample of *C. melanoneura* was higher on apple shoots than on hawthorn shoots (Wald χ^2^ = 10.35; df = 1; *P* = 0.001; Table 3); whereas no host plant effect was observed for the collected-from-hawthorn sample (Wald χ^2^ = 0.05; df = 1; *P* = 0.832; Table 3). No difference in the number of eggs laid during the experiment was recorded between the two *C. melanoneura* samples (*G^2^* = 0.89; df = 1; *P* = 0.344; Fig. 1) or between the different host plants (*G^2^* = 0.94; df = 1; *P* = 0.332; Fig. 1). However, the differences between the oviposition of the two samples are significantly related to the native host plants (*G^2^* = 13.28; df = 1; *P*<0.001; Fig. 1). In particular, the collected-from-apple sample laid more eggs on native shoots (13.74 eggs/day/female) than on hawthorn shoots (0.04 eggs/day/female; Wald χ^2^ = 48.26; df = 1; *P*<0.001) and the collected-from-hawthorn females laid more eggs on hawthorn shoots (13.30 eggs/day/female) than on apple shoots (1 eggs/day/female; Wald χ^2^ = 20.24; df = 1; *P*<0.001). Eggs hatched only on their native host plants as shown in Fig. 2, and no significant differences were found for survival to adulthood of individuals placed on their native host plant shoots (*G^2^* = 0.81; df = 1; *P* = 0.36).

**Table 3:** Survival of remigrant *Cacopsylla melanoneura* females of the two samples on the potential host plants.

Sample	Potential host plant	LT_50_±SE (days)	
ApOL	apple	9.11±2.27	a
	hawthorn	1.90±0.45	b
HaCL	apple	4.00±0.97	a
	hawthorn	4.30±1.03	a

Median lethal times (LT_50_) ± standard error (SE) (in days) are reported. Different letters within a sample indicate significant differences according to the Wald chi-square test (α = 0.05). For the sample acronyms see Table 1.

### Population Genetics Analyses

#### Genetic diversity, Hardy-Weinberg equilibrium and linkage disequilibrium

A total of 577 individuals were genotyped for seven microsatellite loci that proved to be polymorphic in the 16 samples analysed. The number of alleles (N_a_) per locus varied between 3 for locus Co18 (HaRU) and 43 for locus Co12 (CoSO). Allelic richness (*A*_R_) ranged from a minimum value of 2.905 for locus Co18 (HaRU) to a maximum of 24.383 for locus Co12 (CoVA) (Table S1). The average *H*_E_ and *H_O_* ranged from 0.7814 to 0.9575 and from 0.5154 to 0.7094, respectively (Table S1). Loci were not to be in linkage disequilibrium (*P*>0.05, data not shown).

A significant deviation from Hardy-Weinberg equilibrium was observed for most of the analyzed loci and populations (Table S1). Departure from HWE was due to heterozygotes deficit at most of loci. MICROCHECKER did not provide indications of allele dropouts or stuttering at any marker in any sample, whereas it identified null alleles at all loci. Departure from HWE remained significant after correction for null alleles with FREENA analysis, suggesting the presence of null alleles at all loci. To avoid a possible bias due to the presence of null alleles on *F*_ST_ estimation, the dataset was corrected using FREENA [23]. However, samples that failed to amplify were <1%, indicating that null homozygotes were not common. For these reasons, we maintained the original dataset for all analyses while cross-checking differentiation results, when appropriate, by comparing outcomes obtained with corrected dataset (with FREENA). Statistics calculated for the original and the corrected dataset provided comparable results (data not reported). Non significant genotypic disequilibrium was found after Bonferroni correction for multiple tests.

#### Population structure

**Figure 3:**
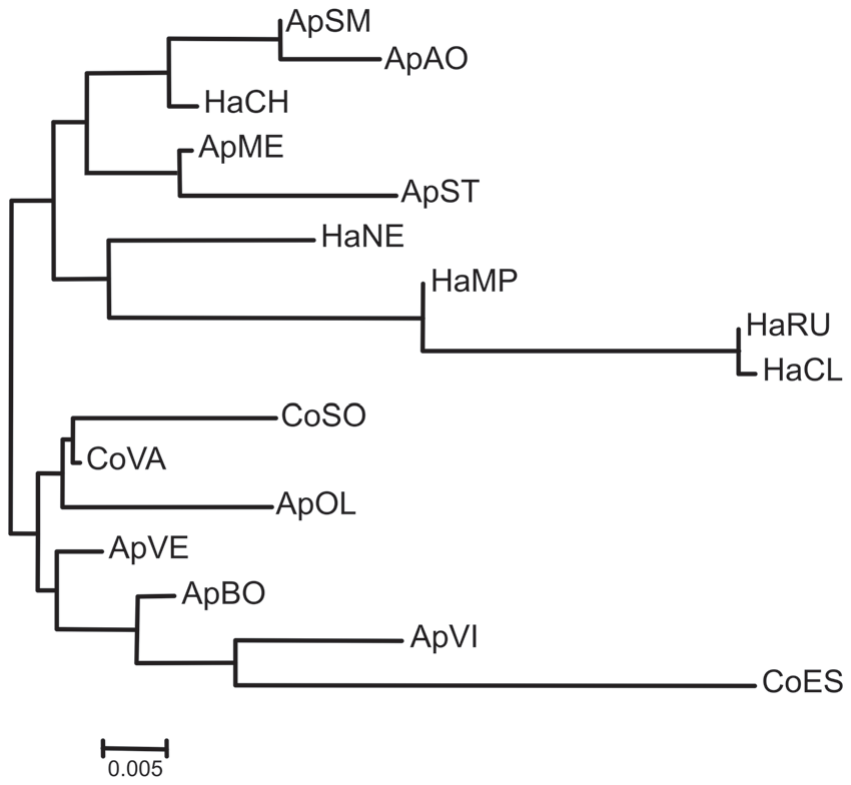
*Cacopsylla melanoneura* population tree. Unrooted Neighbour-Joining population tree based on Slatkin linearized *F*_ST_ pairwise matrix.

Overall *F*_ST_ value was highly significant (*F*_ST = _0.04; 95% Confidence Interval *C.I*. = 0.017 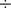 0.039; *P*<0.001) and pair-wise *F*_ST_ revealed significant values for 107 out of 120 comparisons, after Bonferroni correction (Table 4). No differences were found among collected-from-hawthorn samples from Northwestern Italy (HaCL, HaMP, HaRU; Table 4), while significant differences emerged among collected-from-apple samples (Fig. 3; Table 4). When the whole data set was considered, the results obtained indicated that most of the comparisons were significantly different, with some exceptions (Fig. 3; Table 4). The matrix of Slatkin’s linearized *F*_ST_ values is reported in Table S2.

**Table 4:** Genetic differentiation between *Cacopsylla melanoneura* samples.

	ApBO	ApOL	ApSM	ApVE	ApVI	ApAO	ApME	ApST	HaCL	HaMP	HaRU	HaCH	HaNE	CoSO	CoVA	CoES
ApBO		**0.00042**	**0.00042**	0.00292	0.09375	**0.00042**	**0.00042**	**0.00042**	**0.00042**	**0.00042**	**0.00042**	**0.00042**	**0.00042**	**0.00042**	**0.00042**	**0.00042**
ApOL	0.0273		0.00167	0.12167	**0.00042**	**0.00042**	**0.00042**	**0.00042**	**0.00042**	**0.00042**	**0.00042**	**0.00042**	**0.00042**	**0.00042**	0.01042	**0.00042**
ApSM	0.0324	0.0322		0.00292	**0.00042**	0.00125	**0.00042**	**0.00042**	**0.00042**	**0.00042**	**0.00042**	**0.00042**	**0.00042**	**0.00042**	**0.00042**	**0.00042**
ApVE	0.0107	0.0108	0.0159		**0.00042**	**0.00042**	**0.00042**	**0.00042**	**0.00042**	**0.00042**	**0.00042**	**0.00042**	**0.00042**	**0.00042**	**0.00042**	**0.00042**
ApVI	0.0102	0.0499	0.0465	0.0350		**0.00042**	**0.00042**	**0.00042**	**0.00042**	**0.00042**	**0.00042**	**0.00042**	**0.00042**	**0.00042**	**0.00042**	**0.00042**
ApAO	0.0363	0.0445	0.0063	0.0273	0.0526		**0.00042**	**0.00042**	**0.00042**	**0.00042**	**0.00042**	**0.00042**	**0.00042**	**0.00042**	**0.00042**	**0.00042**
ApME	0.0302	0.0414	0.0185	0.0296	0.0430	0.0256		**0.00042**	**0.00042**	**0.00042**	**0.00042**	**0.00042**	**0.00042**	**0.00042**	**0.00042**	**0.00042**
ApST	0.0398	0.0557	0.0266	0.0444	0.0464	0.0309	0.0137		**0.00042**	**0.00042**	**0.00042**	**0.00042**	**0.00042**	**0.00042**	**0.00042**	**0.00042**
HaCL	0.0567	0.0732	0.0692	0.0618	0.0835	0.0833	0.0339	0.0803		0.30417	0.99667	**0.00042**	**0.00042**	**0.00042**	**0.00042**	**0.00042**
HaMP	0.0215	0.0434	0.0369	0.0276	0.0353	0.0459	0.0170	0.0469	0.0101		0.79667	0.00250	**0.00042**	**0.00042**	**0.00042**	**0.00042**
HaRU	0.0617	0.0797	0.0712	0.0649	0.0861	0.0845	0.0368	0.0809	−0.0027	0.0066		**0.00042**	**0.00042**	**0.00042**	**0.00042**	**0.00042**
HaCH	0.0230	0.0421	0.0095	0.0244	0.0398	0.0152	0.0189	0.0329	0.0458	0.0195	0.0451		**0.00042**	**0.00042**	**0.00042**	**0.00042**
HaNE	0.0334	0.0469	0.0335	0.0331	0.0509	0.0382	0.0258	0.0436	0.0555	0.0265	0.0519	0.0269		**0.00042**	**0.00042**	**0.00042**
CoSO	0.0278	0.0394	0.0354	0.0262	0.0478	0.0497	0.0309	0.0463	0.0555	0.0290	0.0576	0.0356	0.0374		0.42708	**0.00042**
CoVA	0.0090	0.0223	0.0176	0.0134	0.0173	0.0259	0.0159	0.0209	0.0614	0.0224	0.0634	0.0212	0.0267	0.0106		0.00583
CoES	0.0492	0.0747	0.0721	0.0577	0.0478	0.0773	0.0664	0.0587	0.1101	0.0610	0.1084	0.0639	0.0736	0.0700	0.0430	

Pair-wise genetic differentiation (*F*_ST_) between *Cacopsylla melanoneura* samples (below diagonal) and associated *P*-values (above diagonal) calculated from the original dataset. Significant *P* values after Bonferroni correction are in bold.

The AMOVA analysis of the samples collected from the two host plants revealed that in Trentino region the population is divided into two groups (collected-from-apple and collected-from-hawthorn) and accounts for 5.21% of the whole variability (*F*_TC = _0.0521; *P* = 0.0169). Moreover, variation among samples within groups and variation among individuals within samples were highly significant (variation rates of 2.55% and 92.24%; *F*_SC = _0.02685 and *F*_ST = _0.07755, respectively; both *P*<0.001). In a second analysis, other samples collected from apple and hawthorn in other regions were added to the data set: the subdivision into two groups was confirmed (*F*_TC = _0.01743; *P* = 0.0386).

**Figure 4:**
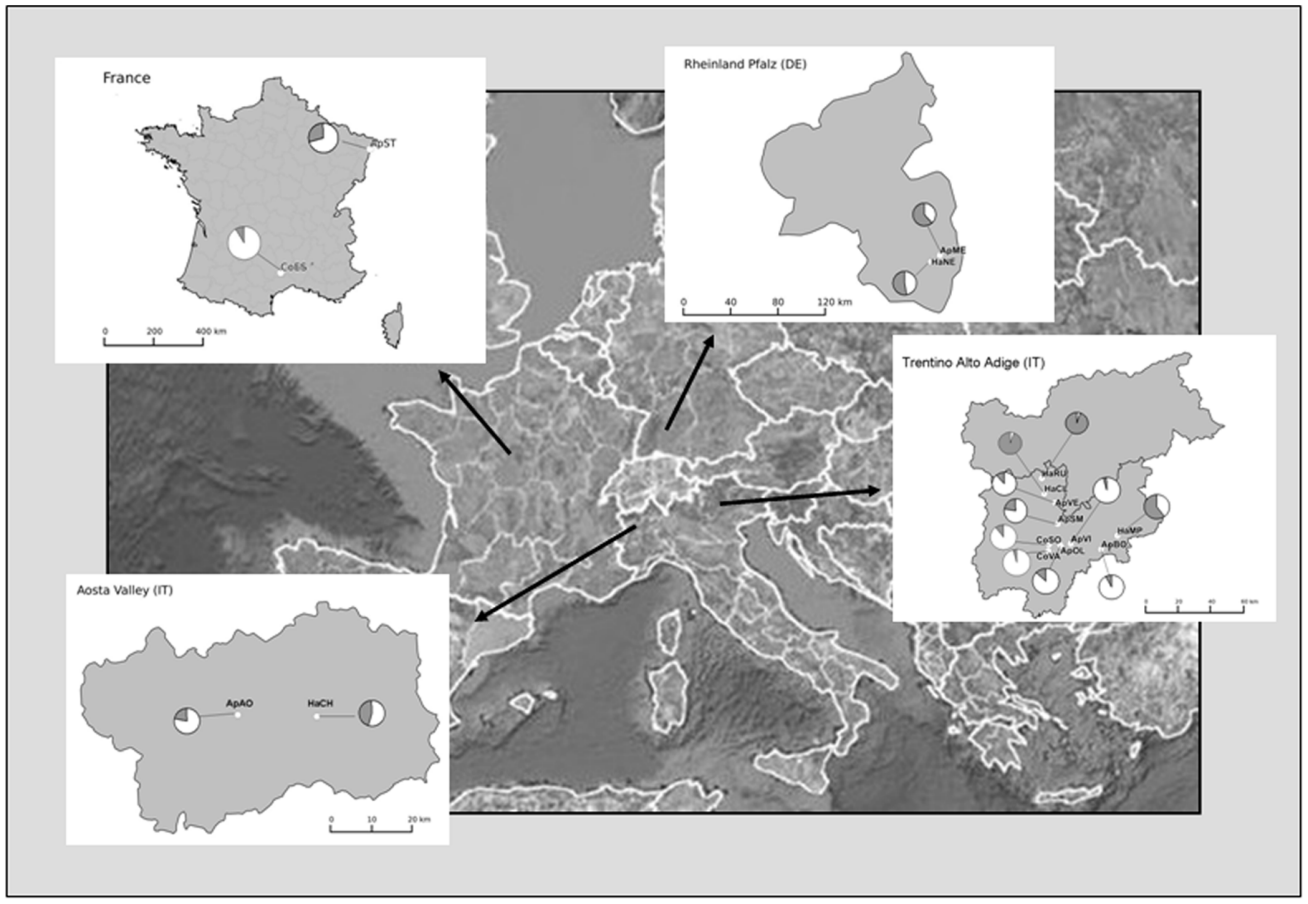
Map of the sampling sites. Insets show detailed maps of Trentino-Alto Adige (Northeastern Italy), Aosta Valley (Northwestern Italy), Rheinland-Pfalz (Southern Germany) and Hesperault (France). The graphs represent the ratio of cluster 1 (white), corresponding to individuals collected from apple with posterior probability greater than 0.70, and cluster 2 (grey), corresponding to individuals collected from hawthorn with posterior probability greater than 0.60, for each sample obtained by STRUCTURE analysis. For samples’ acronyms, see Table 1.

Based on the analysis of data set of Trentino region using structure software, the best model of the number of genetic clusters based on microsatellite variation, as determined by Δ*K* (Evanno’s method), was *K* = 2. All individuals collected from apple plants were assigned to cluster 1 with posterior probability greater than 0.70, while all individuals collected from hawthorn were assigned to cluster 2 with posterior probability major than 0.60. The presence of two main genetic clusters was inferred for the whole dataset with the exception of ApME individuals, which were assigned to the collected-from-hawthorn cluster (Fig. 4).

## Discussion

The ecological advantage for insect host races is widely reported in the literature [36]–[41]. By definition, “host races” are genetically differentiated, sympatric populations of parasites associated with different hosts plant species, on which they feed and reproduce, and between which there is appreciable gene flow [42]. An example of a sympatric host race formation is given in the recent study conducted on *Rhagoletis pomonella* (Walsh), in which a shift from hawthorn to apple is hypothesized [43].

The experiments performed in this study measured the survival and reproductive performance of two *C. melanoneura* groups, showing a significant relationship between this species and its potential host plant. Moreover, a differentiation between the two groups, which are linked to different host plants for their development and reproduction, was suggested by the genetic analyses performed on the different samples.

In the past, *C. melanoneura* was considered to be widely oligophagous on *Crataegus* spp., as in Moravia and Central Europe this species has been observed only on hawthorn [44]. However, there is evidence that *C. melanoneura* lives on, and causes damage to, apple trees (*Malus* spp.) and occasionally to other *Rosaceae* species, such as pear (*Pyrus* spp.) and medlar (*Mespilus germanica* L.) [44]. The presence of different populations of *C. melanoneura* was reported by Lazarev [45], [46], who studied and described the so called *C. melanoneura* form *taurica* (“Crimean apple sucker”), which refers to *C. melanoneura* individuals living on apple trees. Members of the Crimean apple sucker do not develop when transferred to hawthorn, and die within a single week after the transfer, without mating and laying eggs [47]. Furthermore, some morphological differences (e.g. forewing, head, antennae and anal segment length) were observed in *C. melanoneura* samples collected from the two different host plants. Therefore, the presence of different food plants, taken in conjunction with these morphological differences which may correspond to food specialization, enabled to distinguish two forms of the species [46]. In the subsequent years, *C. melanoneura* form *taurica* was considered just as a population living on apple trees and as a synonymous with the typical form [48]. Hence, all the following descriptions gave no taxonomic value to this form and referred to *C. melanoneura* [44]. Anyway, also field observations on *C. melanoneura* naturally occurring in apple orchards and hawthorn hedgerows (Malagnini et al., unpublished data) and preliminary host-switching trials [49] conducted in Northeastern Italy suggested the existence of ecological differences between collected-from-apple and collected-from-hawthorn samples.

The data obtained in this work are in agreement with Lazarev’s studies [46], which were focused on the survival of collected-from-apple samples on hawthorn plants. Unfortunately, the Author did not perform the opposite host-switching experiment, so we cannot compare the results. In our trials we used *C. melanoneura* samples collected directly from apple and hawthorn plants after remigration. As hypothesized by Mayer et al. [50], psyllids may be conditioned by the plants which they had started feeding on: feeding and oviposition experiences can induce a host preference switch in females. Nevertheless, genetic results obtained by Trentino data set suggest that the behaviour of the collected-from-apple and collected-from-hawthorn samples is not due to a conditioning effect, but rather to the existence of host-plant associated populations.

Although not in the aims of this study, some preliminary evidences, which deserve further specific experimental investigations, prompted for the two *C. melanoneura* populations to be good candidates for host race definition. In particular, heterozygote deficit and positive *F*_IS_ values (data not reported) found through most of the samples analyzed and a host-associated trade-off in females’ fitness, suggested the hypothesis of coexisting genetically differentiated pools with ecological differences in agreement with what reported for other psyllid species, such as *C. pruni* (Scopoli) [51], [52] and *C. chinensis* (Yang and Li) [53]. Therefore, rather than being a result of strong null-alleles pervasiveness, the significant heterozygote deficit observed in our data set may be compatible with the hypothesis of a combined effect of species habits and anthropogenic pressure. In fact, the high inbreeding rates may result from the presence of undetected, differentiated populations with small effective population size (N_e_) coexisting at the same sampling area. A condition when populations are subdivided unequally with regard to allele frequencies, so that random mating only involves a portion of the population although gene flow still partially occurs, is also known as Wahlund effect [54]. In this case the heterozygotes deficiency is detected when the differentiated sub-populations are sampled as a single unit [54]. *C. melanoneura* is not a very motile insect: it is passively transported by the wind and, even if it overwinters on conifers at high altitudes, its transfer is not active [55]–[57]. Therefore it can be hypothesised that individuals form small groups, with low genotype diversity, that are transported by the wind, as a single unit, in a preferential direction. In the same manner, psyllids may be brought back to their native areas by winds blowing in the opposite directions [58]. In addition, if overwintering can take place in apple orchards, as suggested by Mattedi et al. [8], mating choice might be forced and reduced to even fewer individuals.

The phytosanitary measures applied in Northern Italy could have contributed, probably together with other factors (such as climatic conditions), to the dramatic decrease in *C. melanoneura* number recorded in Trentino apple orchards between 2000 and 2006 [5] and therefore to a drop in N_e_ and genotype diversity of local populations. This reduction, which is relatively recent, might not have been equally severe in all localities, but may have exacerbated the occurring natural conditions, leading to the genetic differences recorded in this study. This differentiation supported also the ecological differences in host plant preferential choice. In fact, the AMOVA analysis performed on microsatellite dataset of Trentino region indicated small, but significant genetic differences between the collected-from-apple and collected-from-hawthorn populations (*F*_TC = _0.0133; *P* = 0.037). These results were confirmed when the whole data set was considered. STRUCTURE analysis pointed out the presence of two different clusters corresponding to the two host plants for Trentino region. For the other localities (Germany and Aosta Valley), this subdivision seemed to be not that clear. In Germany, samples collected from apple and from hawthorn resulted to be a mixture of both clusters, with hawthorn cluster as predominant. This result seemed to be in agreement with olfactometer analyses: *C. melanoneura* individuals collected after overwintering on conifers preferred hawthorn to apple [50]. These data reflect the abundance and distribution of the two host plants in Germany, where apple cultivation is not as intensive as in Trentino region, where apple dominates over hawthorn and selective pressure is high and mainly based on insecticides. In Aosta Valley, as in Germany, the two clusters co-occurred in the collected-from-hawthorn samples. The presence of a genetic variability among *C. melanoneura* was already observed also by Tedeschi and Nardi [16]. The Authors isolated two different genetic profiles in the mitochondrial control region of Italian populations collected from apple and from hawthorn. The origin and geographic distribution of such variants is yet to be examined in detail. The two profiles were obtained also in this work when we assessed the identity of *C. melanoneura* (data not reported).

However, this study was mainly focused on Trentino region and only few samples from other regions were considered. Future researches should therefore be carried out, involving measurements of the genetic variation between and within populations of *C. melanoneura*, host association and preferential choice, to confirm these preliminary results. These investigations will help in a better understanding of the entire life cycle of *C. melanoneura*. Moreover, inbreeding trials will be useful also to finally assess all the criteria leading to the definition of host races proposed by Dres and Mallet [42].

Noticeably, our study builds upon the knowledge of one vector of AP phytoplasma since recent investigations pointed out that hawthorn may be an *inoculum* source for the spread of this disease through *C. melanoneura* [12]. Nevertheless, according to ecological and genetic data collected in Trentino, hawthorn and apple clusters are different, indicating no significant exchange between them. If these results were replicated at a larger scale, the actual role of hawthorn as reservoir of ‘*Ca.* P. mali’ could be better pinpointed. Furthermore, the significant differences emerged by comparing several collected-from-apple samples could support the different acquisition and transmission efficiencies in *C. melanoneura* samples collected in different areas [4], [59].

## Supporting Information

Table S1 **Genetic variability at seven microsatellite loci for** ***Cacopsylla melanoneura*** **samples.**
(DOC)

Table S2 **Matrix of Slatkin’s linearized FST values for all population samples.**
(DOC)
