# Supplementary material for: Ecological and Genetic Differences between Cacopsylla melanoneura (Hemiptera, Psyllidae) Populations Reveal Species Host Plant Preference
Source: PLoS One. 2013 Jul 16;8(7):e69663. doi: 10.1371/journal.pone.0069663 (PMC3712957; doi:10.1371/journal.pone.0069663)
Supplement: Table S1 — Genetic variability at seven microsatellite loci for Cacopsylla melanoneura samples. (DOC) [file pone.0069663.s001.doc]

**Table S1**

**Genetic variability at seven microsatellite loci for *Cacopsylla melanoneura* samples.** The table provides: *H*_e_, expected heterozygosity; *H*_o_, observed heterozygosity; *P*_HW_, probability of Hardy-Weinberg equilibrium; *A*_R_, allelic richness per locus and population based on the smallest sample size; *Na*, number of alleles for each sample; *S_R_*, allele size (bp) in the original dataset.

| **sample** |  | **locus Co03** |  | **locus Co04** |  | **locus Co11** |  | **locus Co12** |  | **locus Co13** |  | **locus Co14** |  | **locus Co18** |  |
| --- | --- | --- | --- | --- | --- | --- | --- | --- | --- | --- | --- | --- | --- | --- | --- |
| ApBO | *H*_o_ *H*_e_ | 0.9313 | 0.1395 | 0.9673 | 0.2917 | 0.923 | 0.4167 | 0.9754 | 0.3125 | 0.9684 | 0.3750 | 0.9732 | 0.2708 | 0.9002 | 0.1042 |
|  | *P*-val | <0.0001* |  | <0.0001* |  | <0.0001* |  | <0.0001* |  | 0.0052 |  | <0.0001* |  | <0.0001* |  |
|  | *A*_R_ | 17.841 |  | 19.700 |  | 13.369 |  | 21.312 |  | 19.707 |  | 21.294 |  | 12.011 |  |
|  | *Na* | 28 |  | 33 |  | 19 |  | 36 |  | 32 |  | 39 |  | 16 |  |
|  | *S_R_* | 118-298 |  | 70-164 |  | 134-211 |  | 135-249 |  | 123-193 |  | 152-292 |  | 69-89 |  |
| ApOL | *H*_o_ *H*_e_ | 0.9427 | 0.8077 | 0.8706 | 0.3158 | 0.9131 | 0.5 | 0.9388 | 0.3333 | 0.8929 | 0.2308 | 0.9638 | 0.6538 | 0.8653 | 0.1429 |
|  | *P*-val | 0.0029 |  | <0.0001* |  | <0.0001* |  | <0.0001* |  | <0.0001* |  | <0.0001* |  | <0.0001* |  |
|  | *A*_R_ | 14.908 |  | 9.759 |  | 11.707 |  | 13.539 |  | 11.816 |  | 18.794 |  | 7.838 |  |
|  | *Na* | 18 |  | 10 |  | 13 |  | 15 |  | 13 |  | 24 |  | 8 |  |
|  | *S_R_* | 126-238 |  | 86-148 |  | 162-206 |  | 135-247 |  | 129-187 |  | 168-290 |  | 79-127 |  |
| ApSM | *H*_o_ *H*_e_ | 0.9763 | 0.6 | 0.934 | 0.3684 | 0.9372 | 0.6579 | 0.9681 | 0.6098 | 0.9627 | 0.4359 | 0.9746 | 0.5333 | 0.7371 | 0.561 |
|  | *P*-val | <0.0001* |  | <0.0001* |  | <0.0001* |  | 0.0054 |  | <0.0001* |  | <0.0001* |  | <0.0001* |  |
|  | *A*_R_ | 21.502 |  | 16.684 |  | 14.425 |  | 20.627 |  | 18.412 |  | 21.516 |  | 10.749 |  |
|  | *Na* | 31 |  | 25 |  | 21 |  | 37 |  | 26 |  | 33 |  | 16 |  |
|  | *S_R_* | 126-217 |  | 70-130 |  | 134-291 |  | 121-249 |  | 121-201 |  | 156-292 |  | 64-198 |  |
| ApVE | *H*_o_ *H*_e_ | 0.9683 | 0.2308 | 0.9602 | 0.3158 | 0.9406 | 0.5926 | 0.9653 | 0.5000 | 0.9546 | 0.2609 | 0.9495 | 0.125 | 0.8918 | 0.1304 |
|  | *P*-val | <0.0001* |  | 0.0019 |  | <0.0001* |  | 0.0001* |  | <0.0001* |  | <0.0001* |  | <0.0001* |  |
|  | *A*_R_ | 18.857 |  | 16.744 |  | 15.159 |  | 18.830 |  | 17.479 |  | 18.075 |  | 10.292 |  |
|  | *Na* | 23 |  | 18 |  | 19 |  | 24 |  | 21 |  | 22 |  | 11 |  |
|  | *S_R_* | 118-244 |  | 80-146 |  | 139-211 |  | 135-270 |  | 131-213 |  | 115-282 |  | 80-198 |  |
| ApVI | *H*_o_ *H*_e_ | 0.9579 | 0.6 | 0.9513 | 0.25 | 0.9237 | 0.45 | 0.9722 | 0.3 | 0.9718 | 0.4722 | 0.9772 | 0.275 | 0.6239 | 0.2368 |
|  | *P*-val | <0.0001* |  | <0.0001* |  | <0.0001* |  | 0.0184 |  | 0.0014 |  | <0.0001* |  | <0.0001* |  |
|  | *A*_R_ | 19.021 |  | 16.195 |  | 14.392 |  | 20.743 |  | 20.510 |  | 21.868 |  | 8.440 |  |
|  | *Na* | 32 |  | 22 |  | 21 |  | 34 |  | 31 |  | 36 |  | 11 |  |
|  | *S_R_* | 126-240 |  | 80-148 |  | 162-208 |  | 143-266 |  | 123-193 |  | 152-292 |  | 71-87 |  |
| ApAO | *H*_o_ *H*_e_ | 0.9548 | 0.6765 | 0.9018 | 0.6111 | 0.9187 | 0.5 | 0.9731 | 0.6857 | 0.9594 | 0.5152 | 0.9847 | 0.6667 | 0.6451 | 0.6389 |
|  | *P*-val | <0.0001* |  | <0.0001* |  | <0.0001* |  | <0.0001* |  | <0.0001* |  | 0.0534 |  | 0.2198 |  |
|  | *A*_R_ | 18.663 |  | 12.153 |  | 14.373 |  | 21.156 |  | 18.651 |  | 23.949 |  | 8.787 |  |
|  | *Na* | 29 |  | 17 |  | 20 |  | 33 |  | 28 |  | 41 |  | 14 |  |
|  | *S_R_* | 122-230 |  | 74-126 |  | 134-200 |  | 121-243 |  | 123-191 |  | 152-292 |  | 93-145 |  |
| ApME | *H*_o_ *H*_e_ | 0.9456 | 0.7021 | 0.8834 | 0.5918 | 0.9446 | 0.4348 | 0.9508 | 0.6531 | 0.9703 | 0.6122 | 0.9634 | 0.5714 | 0.8336 | 0.9184 |
|  | *P*-val | <0.0001* |  | <0.0001* |  | <0.0001* |  | <0.0001* |  | 0.0104 |  | 0.0242 |  | 0.9383 |  |
|  | *A*_R_ | 16.485 |  | 11.909 |  | 15.987 |  | 18.426 |  | 20.072 |  | 19.997 |  | 8.725 |  |
|  | *Na* | 27 |  | 16 |  | 26 |  | 33 |  | 33 |  | 39 |  | 12 |  |
|  | *S_R_* | 120-193 |  | 74-166 |  | 136-206 |  | 122-200 |  | 123-217 |  | 152-282 |  | 75-157 |  |
| ApST | *H*_o_ *H*_e_ | 0.9459 | 0.9091 | 0.8754 | 0.5 | 0.9352 | 0.2381 | 0.8673 | 0.5227 | 0.9636 | 0.5581 | 0.9732 | 0.5349 | 0.7142 | 0.8864 |
|  | *P*-val | 0.6218 |  | <0.0001* |  | 0.0004* |  | 0.0009 |  | 0.0183 |  | <0.0001* |  | 0.9986 |  |
|  | *A*_R_ | 17.723 |  | 11.521 |  | 14.418 |  | 14.774 |  | 19.207 |  | 21.149 |  | 7.265 |  |
|  | *Na* | 31 |  | 18 |  | 19 |  | 26 |  | 33 |  | 37 |  | 9 |  |
|  | *S_R_* | 116-215 |  | 76-166 |  | 136-160 |  | 124-222 |  | 123-211 |  | 144-282 |  | 79-157 |  |
| HaCL | *H*_o_ *H*_e_ | 0.9271 | 0.8182 | 0.8467 | 0.4545 | 0.9238 | 0.6667 | 0.9268 | 0.7619 | 0.9006 | 0.9545 | 0.8932 | 0.6364 | 0.6776 | 0.7727 |
|  | *P*-val | 0.0388 |  | <0.0001* |  | 0.004 |  | <0.0001* |  | 0.6676 |  | <0.0001* |  | 0.015 |  |
|  | *A*_R_ | 15.028 |  | 11.804 |  | 16.352 |  | 13.593 |  | 11.856 |  | 16.856 |  | 3.904 |  |
|  | *Na* | 18 |  | 14 |  | 18 |  | 16 |  | 14 |  | 21 |  | 4 |  |
|  | *S_R_* | 127-195 |  | 77-167 |  | 138-168 |  | 126-166 |  | 137-151 |  | 151-267 |  | 75-101 |  |
| HaM*P* | *H*_o_ *H*_e_ | 0.961 | 0.8 | 0.9504 | 0.7407 | 0.9559 | 0.8333 | 0.9638 | 0.7 | 0.9508 | 0.9 | 0.9655 | 0.8276 | 0.8277 | 0.8333 |
|  | *P*-val | 0.0022 |  | 0.0039 |  | 0.1101 |  | <0.0001* |  | 0.2098 |  | <0.0001* |  | 0.0335 |  |
|  | *A*_R_ | 19.489 |  | 19.282 |  | 17.235 |  | 19.075 |  | 16.978 |  | 19.981 |  | 8.878 |  |
|  | *Na* | 29 |  | 29 |  | 23 |  | 27 |  | 22 |  | 29 |  | 12 |  |
|  | *S_R_* | 126-216 |  | 72-170 |  | 137-211 |  | 137-216 |  | 135-203 |  | 140-264 |  | 75-133 |  |
| HaRU | *H*_o_ *H*_e_ | 0.9484 | 0.875 | 0.8928 | 0.6341 | 0.9633 | 0.878 | 0.9693 | 0.75 | 0.8949 | 0.9268 | 0.9223 | 0.9024 | 0.5616 | 0.9756 |
|  | *P*-val | 0.125 |  | <0.0001* |  | 0.0012 |  | <0.0001* |  | 0.0192 |  | 0.1166 |  | 0.9997 |  |
|  | *A*_R_ | 17.649 |  | 14.962 |  | 18.891 |  | 20.262 |  | 14.034 |  | 16.727 |  | 2.905 |  |
|  | *Na* | 30 |  | 27 |  | 30 |  | 33 |  | 25 |  | 27 |  | 3 |  |
|  | *S_R_* | 125-195 |  | 72-166 |  | 136-202 |  | 120-200 |  | 123-205 |  | 150-244 |  | 73-103 |  |
| HaCH | *H*_o_ *H*_e_ | 0.9488 | 0.6829 | 0.8964 | 0.5 | 0.9497 | 0.475 | 0.9593 | 0.7857 | 0.9618 | 0.7561 | 0.9727 | 0.7143 | 0.856 | 0.7619 |
|  | *P*-val | <0.0001* |  | 0.0183 |  | <0.0001* |  | <0.0001* |  | 0.0054 |  | <0.0001* |  | <0.0001* |  |
|  | *A*_R_ | 17.979 |  | 15.488 |  | 17.297 |  | 20.516 |  | 18.508 |  | 21.570 |  | 11.479 |  |
|  | *Na* | 31 |  | 27 |  | 26 |  | 38 |  | 29 |  | 41 |  | 17 |  |
|  | *S_R_* | 120-298 |  | 70-166 |  | 134-146 |  | 122-296 |  | 121-203 |  | 150-298 |  | 69-145 |  |
| HaNE | *H*_o_ *H*_e_ | 0.9653 | 0.8889 | 0.96 | 0.5682 | 0.8162 | 0.5778 | 0.9698 | 0.8 | 0.963 | 0.9333 | 0.9436 | 0.5217 | 0.9042 | 0.7292 |
|  | *P*-val | 0.0212 |  | <0.0001* |  | <0.0001* |  | 0.085 |  | 0.5595 |  | <0.0001* |  | 0.0036 |  |
|  | *A*_R_ | 19.378 |  | 19.127 |  | 13.969 |  | 20.343 |  | 19.491 |  | 18.155 |  | 11.731 |  |
|  | *Na* | 33 |  | 34 |  | 28 |  | 36 |  | 37 |  | 34 |  | 16 |  |
|  | *S_R_* | 102-194 |  | 72-170 |  | 130-200 |  | 121-231 |  | 122-217 |  | 151-229 |  | 75-89 |  |
| CoSO | *H*_o_ *H*_e_ | 0.9794 | 0.95 | 0.9817 | 0.7949 | 0.9411 | 0.3846 | 0.9744 | 0.9 | 0.9613 | 0.2424 | 0.9706 | 0.7059 | 0.8095 | 0.7 |
|  | *P*-val | 0.0326 |  | 0.0179 |  | <0.0001* |  | 0.0415 |  | 0.0067 |  | <0.0001* |  | <0.0001* |  |
|  | *A*_R_ | 22.717 |  | 23.327 |  | 16.308 |  | 21.597 |  | 17.630 |  | 20.093 |  | 9.541 |  |
|  | *Na* | 42 |  | 43 |  | 26 |  | 39 |  | 23 |  | 29 |  | 13 |  |
|  | *S_R_* | 118-250 |  | 89-269 |  | 136-266 |  | 124-296 |  | 133-221 |  | 134-298 |  | 75-133 |  |
| CoVA | *H*_o_ *H*_e_ | 0.9671 | 0.8696 | 0.9816 | 0.7826 | 0.9387 | 0.6818 | 0.9865 | 0.7826 | 0.9704 | 0.7727 | 0.9791 | 0.7619 | 0.8744 | 0.8696 |
|  | *P*-val | 0.0214 |  | 0.0008 |  | <0.0001* |  | <0.0001* |  | <0.0001* |  | 0.0136 |  | 0.3401 |  |
|  | *A*_R_ | 20.537 |  | 23.451 |  | 17.548 |  | 24.383 |  | 19.787 |  | 22.076 |  | 11.363 |  |
|  | *Na* | 27 |  | 32 |  | 22 |  | 33 |  | 24 |  | 27 |  | 14 |  |
|  | *S_R_* | 118-244 |  | 77-247 |  | 146-266 |  | 119-296 |  | 131-219 |  | 134-298 |  | 75-133 |  |
| CoES | *H*_o_ *H*_e_ | 0.931 | 0.8 | 0.6501 | 0.5263 | 0.9388 | 0.6316 | 0.9587 | 0.8947 | 0.9333 | 0.6111 | 0.8185 | 0.1875 | 0.7795 | 0.4211 |
|  | *P*-val | 0.0169 |  | 0.0907 |  | <0.0001* |  | 0.0172 |  | <0.0001* |  | <0.0001* |  | <0.0001* |  |
|  | *A*_R_ | 15.000 |  | 7.348 |  | 14.561 |  | 17.812 |  | 15.804 |  | 10.800 |  | 10.025 |  |
|  | *Na* | 15 |  | 8 |  | 16 |  | 20 |  | 17 |  | 11 |  | 11 |  |
|  | *S_R_* | 120-190 |  | 70-160 |  | 138-266 |  | 122-294 |  | 121-203 |  | 152-246 |  | 75-121 |  |
| whole sample** | *H*_o_ *H*_e_ | 0.9532 | 0.7094 | 0.9065 | 0.5154 | 0.929 | 0.5574 | 0.9575 | 0.6433 | 0.9487 | 0.5973 | 0.9516 | 0.5555 | 0.7814 | 0.6052 |
|  | S.D. | ±0.016 | ±0.23 | ±0.081 | ±0.175 | ±0.033 | ±0.166 | ±0.028 | ±0.197 | ±0.028 | ±0.254 | ±0.043 | ±0.230 | ±0.1087 | ±0.303 |
|  | *P*-val | <0.0001* |  | <0.0001* |  | <0.0001* |  | <0.0001* |  | <0.0001* |  | <0.0001* |  | <0.0001* |  |
|  | *A*_R_ | 20.766 |  | 20.953 |  | 18.200 |  | 22.292 |  | 20.131 |  | 22.691 |  | 13.366 |  |
|  | *Na* | 70 |  | 78 |  | 51 |  | 79 |  | 48 |  | 81 |  | 40 |  |

* Significant values after Bonferroni correction

** mean values, S.D. standard deviation
