# Supplementary material for: Ecological and Genetic Differences between Cacopsylla melanoneura (Hemiptera, Psyllidae) Populations Reveal Species Host Plant Preference
Source: PLoS One. 2013 Jul 16;8(7):e69663. doi: 10.1371/journal.pone.0069663 (PMC3712957; doi:10.1371/journal.pone.0069663)
Supplement: Table S2 — Matrix of Slatkin’s linearized FST values for all population samples. (DOC) [file pone.0069663.s002.doc]

**Table S2**

**Matrix of Slatkin’s linearized *F*_ST_ values for all population samples.**

|  | ApBO | ApOL | ApSM | ApVE | ApVI | ApAO | ApME | ApST | HaCL | HaMP | HaRU | HaCH | HaNE | CoSO | CoVA | CoES |
| --- | --- | --- | --- | --- | --- | --- | --- | --- | --- | --- | --- | --- | --- | --- | --- | --- |
| ApBO |  | 0.02397 | 0.03465 | 0.00500 | 0.01532 | 0.04462 | 0.03661 | 0.04777 | 0.03825 | 0.02941 | 0.07098 | 0.02724 | 0.06821 | 0.03105 | 0.01671 | 0.05973 |
| ApOL |  |  | 0.02469 | 0.02195 | 0.04862 | 0.04425 | 0.03670 | 0.05441 | 0.04631 | 0.03789 | 0.07798 | 0.04213 | 0.08005 | 0.03278 | 0.01766 | 0.07619 |
| ApSM |  |  |  | 0.01462 | 0.04657 | 0.00244 | 0.01498 | 0.02352 | 0.03325 | 0.00463 | 0.07685 | 0.03757 | 0.08070 | 0.03812 | 0.01833 | 0.09559 |
| ApVE |  |  |  |  | 0.03426 | 0.02657 | 0.02121 | 0.04139 | 0.03198 | 0.01709 | 0.06123 | 0.02551 | 0.06726 | 0.02431 | 0.00991 | 0.06990 |
| ApVI |  |  |  |  |  | 0.06162 | 0.04829 | 0.05385 | 0.05678 | 0.04545 | 0.09709 | 0.04032 | 0.09746 | 0.05498 | 0.02399 | 0.05370 |
| ApAO |  |  |  |  |  |  | 0.03085 | 0.03674 | 0.04359 | 0.01938 | 0.09683 | 0.05201 | 0.10176 | 0.05753 | 0.03186 | 0.09018 |
| ApME |  |  |  |  |  |  |  | 0.01770 | 0.02819 | 0.02299 | 0.04092 | 0.02032 | 0.03937 | 0.03308 | 0.02048 | 0.07202 |
| ApST |  |  |  |  |  |  |  |  | 0.04891 | 0.03854 | 0.09197 | 0.05389 | 0.09387 | 0.05253 | 0.02691 | 0.06218 |
| HaCL |  |  |  |  |  |  |  |  |  | 0.02970 | 0.05759 | 0.02993 | 0.06361 | 0.04103 | 0.03025 | 0.08319 |
| HaMP |  |  |  |  |  |  |  |  |  |  | 0.05013 | 0.02279 | 0.05228 | 0.03913 | 0.02581 | 0.06902 |
| HaRU |  |  |  |  |  |  |  |  |  |  |  | 0.00787 | 0.00000 | 0.06279 | 0.07013 | 0.11943 |
| HaCH |  |  |  |  |  |  |  |  |  |  |  |  | 0.01104 | 0.03172 | 0.02548 | 0.06356 |
| HaNE |  |  |  |  |  |  |  |  |  |  |  |  |  | 0.06286 | 0.07066 | 0.12807 |
| CoSO |  |  |  |  |  |  |  |  |  |  |  |  |  |  | 0.01611 | 0.08080 |
| CoVA |  |  |  |  |  |  |  |  |  |  |  |  |  |  |  | 0.04706 |
| CoES |  |  |  |  |  |  |  |  |  |  |  |  |  |  |  |  |
